# Increased Tissue Penetration of Doxorubicin in Pressurized Intraperitoneal Aerosol Chemotherapy (PIPAC) after High-Intensity Ultrasound (HIUS)

**DOI:** 10.1155/2019/6185313

**Published:** 2019-12-12

**Authors:** Veria Khosrawipour, Sören Reinhard, Alice Martino, Tanja Khosrawipour, Mohamed Arafkas, Agata Mikolajczyk

**Affiliations:** ^1^Division of Colorectal Surgery, Department of Surgery, University of California Irvine (UCI), Irvine, CA, USA; ^2^Department of Bioengineering, University of California, Berkeley (UC Berkeley), Oakland, CA, USA; ^3^Department of Surgery, University-Hospital Düsseldorf, Düsseldorf, North-Rhein Westfalia, Germany; ^4^Department of Plastic Surgery, Ortho-Klinik Dortmund, Dortmund, North-Rhein Westfalia, Germany; ^5^Department of Biochemistry and Molecular Biology, Faculty of Veterinary Medicine, Wroclaw University of Environmental and Life Sciences, Wroclaw, Lower Silesia, Poland

## Abstract

**Background:**

High‐intensity ultrasound (HIUS) has been studied for the past two decades as a new therapeutic option for solid tumor direct treatment and a method for better chemotherapy delivery and perfusion. This treatment approach has not been tested to our knowledge in peritoneal metastatic therapy, where limited tissue penetration of intraperitoneal chemotherapy has been a main problem. Both liquid instillations and pressurized aerosols are affected by this limitation. This study was performed to evaluate whether HIUS improves chemotherapy penetration rates.

**Methods:**

High-intensity ultrasound (HIUS) was applied for 0, 5, 30, 60, 120, and 300 seconds on the peritoneal tissue samples from fresh postmortem swine. Samples were then treated with doxorubicin via pressurized intraperitoneal aerosol chemotherapy (PIPAC) under 12 mmHg and 37°C temperature. Tissue penetration of doxorubicin was measured using fluorescence microscopy on frozen thin sections.

**Results:**

Macroscopic structural changes, identified by swelling of the superficial layer of the peritoneal surface, were observed after 120 seconds of HIUS. Maximum doxorubicin penetration was significantly higher in peritoneum treated with HIUS for 300 seconds, with a depth of 962.88 ± 161.4 *μ*m (*p* < 0.05). Samples without HIUS had a penetration depth of 252.25 ± 60.41. Tissue penetration was significantly increased with longer HIUS duration, with up to 3.8-fold increased penetration after 300 sec of HIUS treatment.

**Conclusion:**

Our data indicate that HIUS may be used as a method to prepare the peritoneal tissue for intraperitoneal chemotherapy. Higher tissue penetration rates can be achieved without increasing chemotherapy concentrations and preventing structural damage to tissue using short time intervals. More studies need to be performed to analyze the effect of HIUS in combination with intraperitoneal chemotherapy.

## 1. Introduction

Peritoneal metastasis (PM) is a commonly seen manifestation of advanced gastrointestinal and gynecological cancers. It is known that the antitumor effect of intraperitoneal chemotherapy (IPC) is strongly limited by penetration of chemotherapy drugs well below 1 mm into peritoneal nodules [1, 2]. Various approaches have been made to improve the availability of chemotherapy in these tumor nodules.

For example, it has been shown that hyperthermia [3] and intraperitoneal pressure [4] increase drug penetration and efficiency. These concepts have already led to new therapies like hyperthermic intraperitoneal chemotherapy (HIPEC) combined with cytoreductive surgery [5]. The application of pressure has been proposed and was ultimately applied through pressurized intraperitoneal aerosol chemotherapy (PIPAC) in the treatment of more advanced peritoneal metastasis [6, 7]. Clinical as well as experimental studies have also tested irradiation [8–10] and new drug formulas [11–13] as alternate methods of increasing chemotherapy penetration. Despite several attempts to improve penetration rates, these studies have had only limited success. For example, application parameters in PIPAC that may affect chemotherapeutic penetration depth, such as the micropump position and dose of doxorubicin, have been tested [14]. Although many of these aforementioned efforts have been made, penetration levels still were mostly described to be less than 500 *μ*m [15–17].

Therefore, further methods for improved drug delivery and increasing depth penetration are needed to be developed. In this regard, high intensity ultrasound (HIUS) has been a very promising method to potentially achieve this goal. HIUS has been investigated for over two decades in solid tumor therapy with promising results in particular cases [18–20]. It is already known that HIUS can improve the perfusion of chemotherapy agents in liver tumors and glioblastoma [21, 22]. HIUS systems provide unique advantages of low invasiveness and absence of radiation.

However, to our knowledge, its interaction in combination with any form of intraperitoneal chemotherapy on the peritoneum has not been thoroughly investigated. It is known that HIUS enhances the delivery of doxorubicin in a preclinical model of solid pancreatic cancer [23]. We aimed to evaluate its effect on the penetration depth of doxorubicin in a well-established model of fresh postmortem peritoneal tissue samples [17, 24].

## 2. Materials and Methods

### 2.1. High-Intensity Ultrasound

The experiments were performed on commercially available tissue samples; hence, no approval of the Local Board on Animal Care was required. Fresh postmortem swine peritoneum was purchased (local pork supplier, Zerniki Wielkie) and cut into proportional sections. Samples were then placed into Petri dishes. and NaCl 0.9% was added until the peritoneal surface was covered with 5 mm of liquid. High-intensity ultrasound was applied with a metal pen to the center of the peritoneal tissue using a sonicator (Bandelin Sonoplus, UW 2070). The tip of the pen was held 3 mm from the tissue. Samples were divided into six groups which were treated for 0 seconds, 5 seconds, 30 seconds, 60 seconds, 120 seconds, and 300 seconds, respectively. Each treatment contained 0.3 seconds of active and 0.7 seconds of passive interval, with 20 kHz frequency, output power of 70 W, and 50% of amplitude.

### 2.2. *Ex Vivo* PIPAC Model

Samples and untreated controls were placed in a well-described *ex vivo* model and treated with PIPAC with doxorubicin (PFS®, 2 mg/ml, Pfizer Europe, Sandwich, United Kingdom, purity ≥98%). A commercially available hermetic sealable plastic box with a total volume of 3.5 liters, representing the abdominal cavity, was used. In the center of the top cover of the plastic box, a 5 mm trocar (Kii® Balloon Blunt Tip System, Applied Medical, Rancho Santa Margarita, CA, USA) was placed. The nozzle of the microcatheter (MC, Olympus, PW-205V Olympus Surgical Technologies Europe, Hamburg, Germany) was introduced into the trocar. The plastic box was kept at a constant temperature of 27°C during the whole procedure. Fresh tissue specimens of peritoneum (German landrace pigs), each measuring 4.0 × 4.0 × 0.5 cm, were placed at the center of the plastic box. The distance between the nozzle of the MC and the bottom of the plastic box was 10 cm. The plastic box was then tightly sealed, and a constant CO_2_ capnoperitoneum of 12 mmHg (Olympus UHI-3, Olympus medical life science and industrial divisions, Olympus Australia, Notting Hill, Australia) was maintained during the entire PIPAC procedure. 3 mg of doxorubicin were dissolved in 50 ml NaCl 0.9% at 27°C and aerosolized.

### 2.3. Microscopic Analysis

After treatments, all tissue samples were rinsed with a sterile NaCl 0.9% solution in order to eliminate superficial chemotherapy and immediately frozen in liquid nitrogen. Cryosections (10 *μ*m) were prepared from different areas of each specimen. Sections were mounted with a ProLong™ Gold Antifade Mountant (Thermo Fisher Scientific) containing 1.5 *μ*g/ml 4',6-diamidino-2-phenylindole (DAPI) to stain nuclei. Penetration depth of doxorubicin was monitored using a Nikon Eclipse 80i fluorescence microscope (Nikon Instruments Europe B.V. Amsterdam, Netherlands). The distance between the luminal surface and the innermost positive staining for doxorubicin accumulation was measured and reported in micrometers.

### 2.4. Statistical Analyses

Experiments were independently performed three times. A total of eight tissue sections per tissue sample were subject to doxorubicin penetration measurement. Prism 7.0 software (GraphPad, La Jolla, CA, USA) was utilized to analyze the data. One-way ANOVA with a multiple comparison test was used for analyses of independent groups. A significant *p* value was considered at *p* < 0.05.

## 3. Results

### 3.1. *Ex Vivo* Experiment

PIPAC and HIUS were applied without complications. After applying HIUS, a visual control of the sample was performed. No macroscopic damage of the peritoneal surface was observed with shorter HIUS duration. However, after 120 seconds, some whitening and swelling of the peritoneum were noted. Doxorubicin was detected in fluorescence microscopy in both groups. Microscopic analysis of the different tissue specimens showed a substantial difference in the penetration depth of doxorubicin. Tissue penetration levels after HIUS were 361 *μ*m ± 34.5 *μ*m at 5 seconds, 409 *μ*m ± 69.7 *μ*m at 30 seconds, 598 *μ*m ± 136.9 *μ*m at 60 seconds, 725 *μ*m ± 126.4 *μ*m at 120 seconds, and 962 *μ*m ± 161.4 *μ*m at 300 seconds. Controls without HIUS showed penetration levels with (A) 252 *μ*m ± 60.4 *μ*m. Penetration increased significantly with longer HIUS duration (A-F vs. controls, *p* < 0.05) and reached a maximum in the sample (F). The penetration reached the 1 mm level (F) and increased up to 3.8 folds to the control without HIUS (control vs. F, *p* < 0.0001).

**Figure 1:**
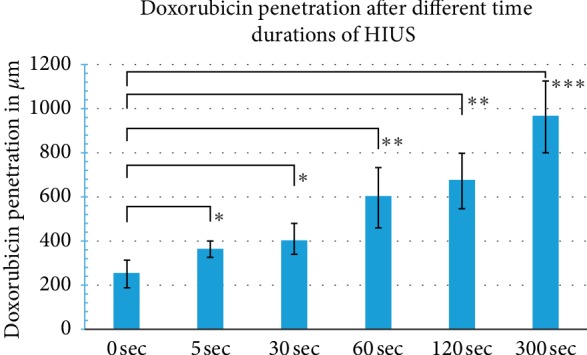
Tissue penetration depth of doxorubicin in *μ*m after HIUS treatment for 0, 5, 30, 60, 120, and 300 sec (^*∗*^*p* < 0.01; ^*∗∗*^*p* < 0.001; ^*∗∗∗*^*p* < 0.0001).

**Figure 2:**
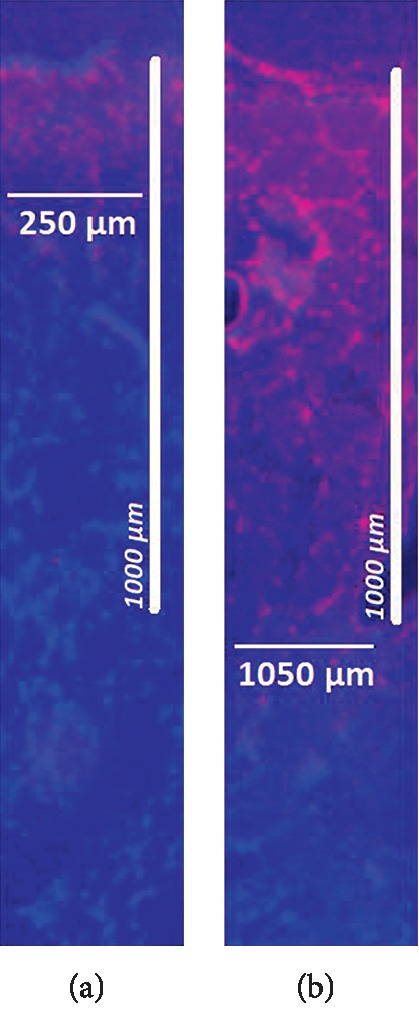
Microscopic analysis of the penetration depth of doxorubicin into fresh peritoneal samples of German landrace pigs. Nuclei (blue) were stained with 4',6-diamindino-2-phenylidole (DAPI). (a) In-tissue penetration of doxorubicin without HIUS. (b) In-tissue penetration of doxorubicin after 300 seconds HIUS.

The differences between the penetration depths observed in this study summarized in Figures 1 and 2 display representative photos showing doxorubicin ﬂuorescence in the analyzed tissue samples.

## 4. Discussion

In spite of progress in chemotherapeutic regimens and new drug compositions, poor response to systemic and local treatment is observed in a considerable part of patients, mainly due to molecular mechanisms and limited drug distribution in the tumor [1, 25].

Pressurized intraperitoneal and pressurized intraluminal aerosol chemotherapies have been introduced to improve the treatment of advanced, multiresistant surface malignancies by overcoming limitations in drug penetration through the use of pressure and microaerosol [26, 27]. However, attempts to further improve were only partially successful, as changes of treatment parameters have only modestly improved penetration rates [4, 14]. Adding irradiation and modifying application modes [10] did not improve performance either, as penetration levels were mostly limited to the first few hundred microns. However, we know that increasing tissue penetration enhances the antitumor effect with a higher local drug disposition [1]. In our study, we demonstrate the previously unrecognized potential of HIUS to enhance drug penetration to many folds in the peritoneal tissue.

In the clinical setting, HIUS is being increasingly used as noninvasive treatment of both primary and metastatic tumors. Besides its effects described here, it has additional antitumor effects including ablation and mechanical disruption of cancer tissue [28, 29]. HIUS has already been shown to be useful in the treatment of uterine fibroids [30], various solid tumors of pancreas, liver, renal system, and prostate, and breast cancer [31–34]. So far, there have been no or few studies for potential use in peritoneal metastases (PM). By improving tissue penetration, higher drug concentrations in the tumor tissue could be reached without increasing the drug dose, which is important to limit systemic side effects of the chemotherapy.

Other attempts to improve current PIPAC and IPC have been studied recently. One such attempt to improve overall results is synchronous intravenous chemotherapy. Feasibility for this kind of bidirectional approach has been demonstrated, and results on tumor regression and survival have been promising [35]. However, it is unclear whether this effect is predominantly that of PIPAC or rather one of the intravenous chemotherapies. Studies indicate that this might be an effect of PIPAC [36], while the effect of the additional intravenous chemotherapy is unknown.

Another attempt to improve PIPAC was the introduction of electrostatic precipitation as an additional feature to the procedure. A recent study from Giger-Pabst et al. [37] analyzing the effect of electrostatic PIPAC (ePIPAC) versus PIPAC alone did not show any tissue increase or any other change demonstrating the efficancy of PIPAC by adding an electrostatic device. Additionally, clinical studies could not detect any differences between these two approaches in terms of biological effect [38]. Data on electrostatic augmentation is scarce, and the potential of electrostatic PIPAC is unknown. Analyzing the effects of electrostatic precipitation combined with the applied aerosol itself is quite a challenge, and while ongoing studies present new locations and various applications for chemoaerosol [39, 40], there is an ongoing effort to understand the applied chemoaerosol itself [39, 41].

The application of heat in IPC is well studied. Heat has shown to increase cytotoxicity and has therefore been an integral part in HIPEC [42]. However, the application of heat in PIPAC is a technical challenge because heat would have to be distributed through the applied gaseous capnoperitoneum. Therefore, it remains unclear if heat has a role in PIPAC. Despite these limitations, concepts based on basic physical principles like heat, electrostatic effects, changing physical properties of applied substances [39], or mechanic alteration [43] of the biological surface have gained more interest recently as they seem to have more potential than initially expected.

Our data indicate that HIUS plus PIPAC can overcome the 1 mm barrier on the peritoneum, which is a very promising result. HIUS resulted in better penetration of doxorubicin into swine peritoneum samples from 1.4 to 3.8 folds depending on the duration of HIUS application (5 sec to 300 sec). These findings require further studies in this field and ideas for a possible clinical approach to the application of HIUS in PM via PIPAC or via any other intraperitoneal chemotherapy.

## 5. Conclusions

Our data indicate that pretreatment of tissue samples with HIUS enhances doxorubicin penetration after the PIPAC procedure. Depth of penetration increases with longer duration of HIUS. This can be a new promising approach in IPC for better outcomes. Further research needs to be conducted for translation of this *ex vivo* method into clinical practice.

## Data Availability

The data used to support the findings of this study are available from the corresponding author on request.

## Abbreviations

CO2: Carbon dioxide
CRS: Cytoreductive surgery
HIPEC: Hyperthermic intraperitoneal chemotherapy
HIUS: High-intensity ultrasounds
IAP: Intra-abdominal pressure
IPC: Intraperitoneal chemotherapy
PM: Peritoneal metastasis
PIPAC: Pressurized intraperitoneal aerosol chemotherapy
ePIPAC: Electrostatic pressurized intraperitoneal aerosol chemotherapy
MC: Microcatheter.
